# 

**Published:** 1882-07

**Authors:** 


### CONTENTS:

ART. I.—Pericementitis. By G. A. Mills, D. D. S. .......... 97
ART. II.—To the Dental Profession .......... 107
ART. III.—Rights and Liabilities of Dentists at Common Law. By Jas. G. Dunning L. L. B. .......... 113
ART. IV.—Vacancies in the Dental Arch By W. C. Wardlaw, D. D. S. .......... 117
ART. V.—Dental Education. By J. B. Hodgkin, D. D. S .......... 120
ART. VI.—The Disorders of Primary Dentition .......... 124
ART. VII.—The Follies of Fashion .......... 128
ART. VIII.—Farinaceous Infant Foods .......... 130
ART. IX.—The American Medical Association .......... 131
Pennsylvania State Dental Society .......... 133
National Dental Association .......... 134
American Dental Association .......... 134
Southern Dental Association .......... 135
New York State Dental Society .......... 135

### EDITORIAL, ETC.

The Connection of Dental with Medical Education .......... 135
Maryland University Dental Department Building .......... 139
The Amalgam Question .......... 140
The Two Editors .......... 142

### MONTHLY SUMMARY.

The Medicinal Value of Vegetables .......... 142
Is this a New Dental Disease? .......... 143
Removal of Foreign Bodies from the Ear .......... 143
Congenital Teeth .......... 144

